# Co_3_O_4_ nanoparticles anchored on nitrogen-doped reduced graphene oxide as a multifunctional catalyst for H_2_O_2_ reduction, oxygen reduction and evolution reaction

**DOI:** 10.1038/srep43638

**Published:** 2017-03-08

**Authors:** Tingting Zhang, Chuansheng He, Fengzhan Sun, Yongqi Ding, Manchao Wang, Lin Peng, Jiahui Wang, Yuqing Lin

**Affiliations:** 1 Department of Chemistry, Capital Normal University, Beijing, 100048, China

## Abstract

This study describes a facile and effective route to synthesize hybrid material consisting of Co_3_O_4_ nanoparticles anchored on nitrogen-doped reduced graphene oxide (Co_3_O_4_/N-rGO) as a high-performance tri-functional catalyst for oxygen reduction reaction (ORR), oxygen evolution reaction (OER) and H_2_O_2_ sensing. Electrocatalytic activity of Co_3_O_4_/N-rGO to hydrogen peroxide reduction was tested by cyclic voltammetry (CV), linear sweep voltammetry (LSV) and chronoamperometry. Under a reduction potential at −0.6 V to H_2_O_2_, this constructing H_2_O_2_ sensor exhibits a linear response ranging from 0.2 to 17.5 mM with a detection limit to be 0.1 mM. Although Co_3_O_4_/rGO or nitrogen-doped reduced graphene oxide (N-rGO) alone has little catalytic activity, the Co_3_O_4_/N-rGO exhibits high ORR activity. The Co_3_O_4_/N-rGO hybrid demonstrates satisfied catalytic activity with ORR peak potential to be −0.26 V (vs. Ag/AgCl) and the number of electron transfer number is 3.4, but superior stability to Pt/C in alkaline solutions. The same hybrid is also highly active for OER with the onset potential, current density and Tafel slope to be better than Pt/C. The unusual catalytic activity of Co_3_O_4_/N-rGO for hydrogen peroxide reduction, ORR and OER may be ascribed to synergetic chemical coupling effects between Co_3_O_4_, nitrogen and graphene.

With the transition from traditional fossil fuels to clean and sustainable energy, lots of attentions have been paid on storage systems with environmental benignity, high efficiency and alternative energy conversion. Fuel cells have been considered as the most efficient and clean energy conversion device because fuels react with oxygen via mild electrochemical processes without combustion and the overall fuel-conversion efficiency is not limited by the Carnot cycle laws^1^,^2^. Designing bifunctional catalysts with good oxygen reduction reaction (ORR) and oxygen evolution reaction (OER) activities would be highly beneficial to the development of metal-air batteries. However, developing catalysts for ORR and OER with high activity at low cost remain great challenges^3^,^4^. Platinum-based materials are known to be the most active electrocatalysts for the ORR and the OER. However, the limited reserves of Pt, high cost, the activity deterioration with time and poor durability severely hinder the large-scale applications of Pt in ORR and OER^5^,^6^,^7^,^8^.

On the other hand, the determination of hydrogen peroxide (H_2_O_2_) has aroused more and more interests of researchers as its significance in the fields of applications in industry as well as biological reactions. Therefore, a rapid, accurate and reliable method to detect H_2_O_2_ is of highly demanded. Among various techniques for H_2_O_2_ detection, electrochemical H_2_O_2_ electrocatalysts are promising due to their high sensitivity, low cost, good selectivity, easy for automation and operational simplicity^9^,^10^,^11^,^12^. Catalysts for hydrogen peroxide reduction, oxygen reduction and oxygen evolution reactions are vital in biological assay and renewable-energy technologies including fuel cells and water splitting.

Recently, transition metal oxides including magnanimous oxide, cobalt oxide, iron oxide and nickel oxide as promising materials have received considerableattention due to their low cost, high abundance and perfect catalytic activity for the ORR, OER and immobilizing enzymes for further applications in fabrication of hydrogen peroxide biosensor^13^,^14^,^15^. Among them, Co_3_O_4_ with spinel crystal structure is beneficial to electron transportation between Co^2+^ and Co^3+^ ions, which has been extensively considered as an efficient electrocatalyst for OER and ORR^16^,^17^,^18^. Previous studies reported that the efficiency of cobalt oxide as an OER catalyst could be ascribed to the increasing population of Co^IV^ centers at the oxide surface during electrochemical oxidation^19^. More interesting, Co_3_O_4_, which exhibits catalase-like activity for the decomposition of H_2_O_2_, can be applied to the detection of H_2_O_2_ in aqueous medium^20^. However, these Co_3_O_4_-based catalysts usually suffer from the poor electrical conductivity, short active site density and the dissolution or agglomeration during electrochemical processes. Co_3_O_4_ itself is a material with a little ORR, OER and H_2_O_2_ sensing activity and further studies exhibit that synergy between the carbon materials and Co_3_O_4_ can give a huge promotion of the electrocatalytic activity^21^,^22^. Therefore, lots of researches have used conductive carbon nanomaterials such as carbon nanotubes (CNTs), carbon foam and graphene etc. To improve the conductivity of Co_3_O_4_ based hybrid catalysts as well as obtain uniformly dispersed Co_3_O_4_ nanoparticles and thus to improve the electrocatalyst activity.

Graphene, a two-dimensional layer framework of sp2-hybridized carbon with outstanding chemical and physical properties, has attracted a lot of attention in the last years^21^,^23^,^24^. Graphene could be an attractive support for metal oxides to form a new class of nanocomposites for ORR due to their notable electronic conductivity and high surface area^25^,^26^. Dai’s group reported a hybrid material consisting of Co_3_O_4_ nanocrystals grown on reduced graphene oxide as a high-performance bi-functional catalyst for the ORR and OER^27^. Wang etc. synthesized a novel multifunctional nanohybrid by chemically coupling ultrafine metal oxide nanoparticles to reduced grapheme oxide (rGO) as an effective catalyst for oxygen reduction reaction^28^. Anchoring Co_3_O_4_ nanocrystals on carbon-based supports could significantly improve their electrocatalytic activity contributed by the small crystalline size and conductive support^29^. What’s more, chemical doping with hetero atoms is an efficacious method to regulate electronic properties and surface chemistry of assembled graphene by the modulation of the carbon-carbon bonds^30^,^31^. It has been also reported that nitrogen-doped graphene can promote the electrochemical reduction of H_2_O_2_^32^. As previous study, the introduction of the Co−N_4_ complex onto the graphene basal plane facilitates the activation of O_2_ dissociation and the desorption of H_2_O during the ORR^33^. Nitrogen-graphene can produce the synergistic support effect because the reactive intermediates such as hydrogen peroxide are known to decomposed by nitrogen doped carbon nanostructures. However, so far, few researches have reported catalyst which has three functions for H_2_O_2_ reduction, ORR and OER.

We report herein the synthesis of Co_3_O_4_ nanoparticles anchored on nitrogen-doped reduced graphene oxide (Co_3_O_4_/N-rGO) through a simple and scalable method as tri-functional catalysts for H_2_O_2_ reduction, ORR and the OER, as shown in Fig. 1. Co_3_O_4_ anchored uniformly into laminar nitrogen-doped reduced graphene oxide was confirmed by scanning electron microscopy (SEM). Co_3_O_4_/N-rGO possesses a good electrocatalytic activity toward H_2_O_2_ reduction by enhancing the current response and decreasing H_2_O_2_ reduction over potential. The electrochemical results demonstrate that the Co_3_O_4_/N-rGO can exhibit higher activity for both the ORR and the OER and better durability than a commercial carbon-supported Pt catalyst. The strong coupling between Co_3_O_4_, nitrogen and reduced graphene oxide (rGO) is found to play an important role in the high electrocatalytic activities of the Co_3_O_4_/N-rGO. This synthesis route can be easily adopted for large-scale manufacturing due to its process simplicity and the accessibility of precursor materials.

## Results and Discussion

### Characterization

**Figure 2:**
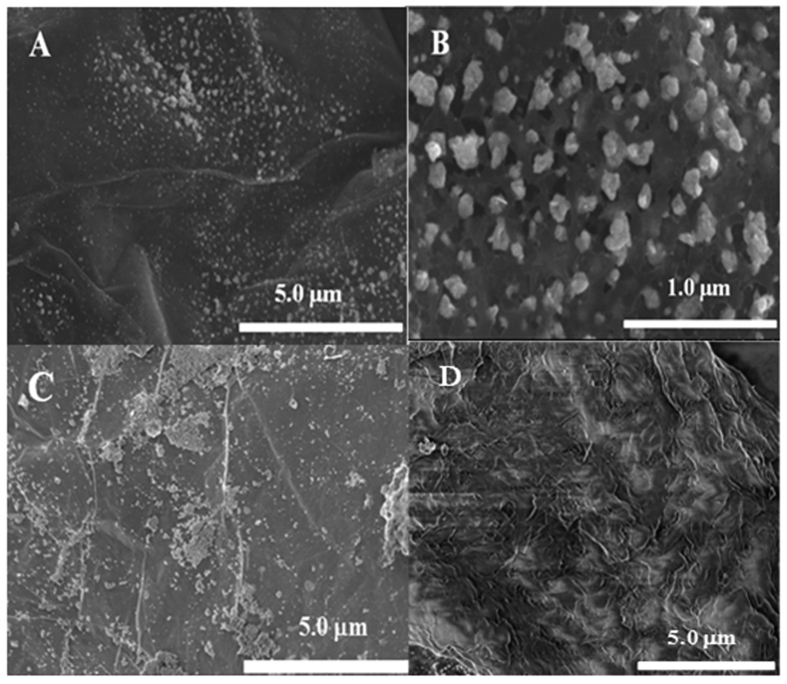
Scanning electron microscopy image of Co_3_O_4_/N-rGO (**A,B**), Co_3_O_4_/rGO (**C**) and N-rGO (**D**), respectively.

**Figure 3:**
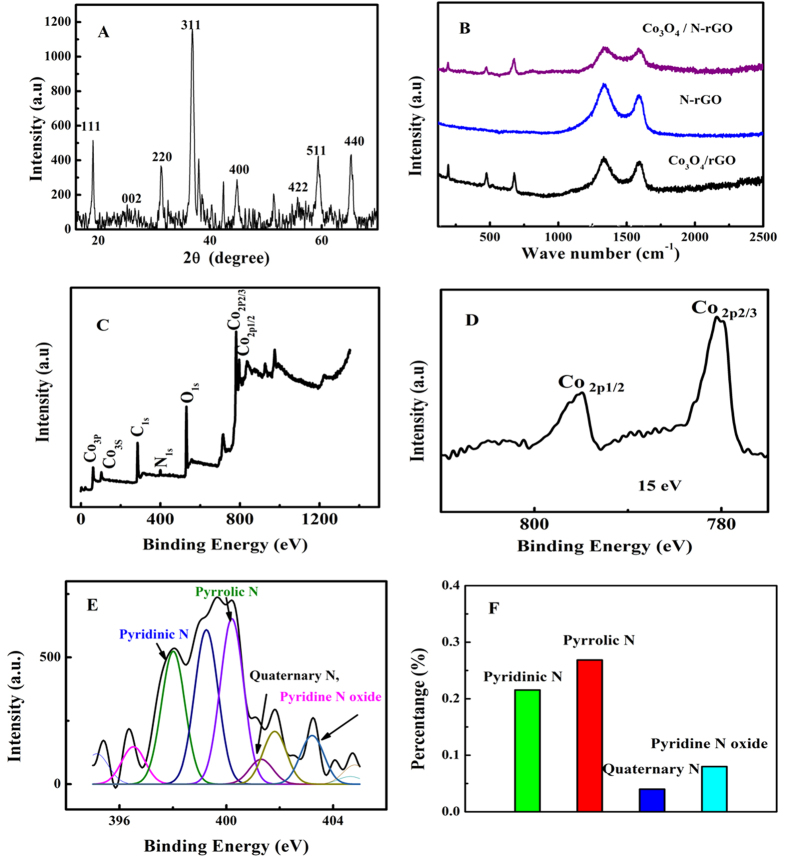
XRD spectrum of Co_3_O_4_/N-rGO (**A**) and Raman spectra of Co_3_O_4_/N-rGO, N-rGO and Co_3_O_4_/rGO (**B**); The XPS full spectrum of Co_3_O_4_/N-rGO (**C**) and high resolution Co_2p_ spectra of Co_3_O_4_/N-rGO (**D**); High-resolution N1s XPS spectra of Co_3_O_4_/N-rGO (**E**); The percentage of three nitrogen species in Co_3_O_4_/N-rGO (E).

Figure 2A and B illustrated field emission scanning electron microscopy (FE-SEM) images of Co_3_O_4_/N-rGO. We can clearly see from SEM images in Fig. 2B that Co_3_O_4_ nanoparticles are uniformly anchored on the rGO substrate with an approximate average diameter of 150 nm. This may be attributed that Co^2+^ ion was coordinated with negatively charged oxygen-containing functional groups on N-rGO sheets. During the hydrothermal process, Co^2+^ was oxidized into Co^3+^ by oxygen-containing groups, and crystallized to form Co_3_O_4_ nanoparticles anchored into N-rGO sheets^34^. However, Fig. 2C demonstrate Co_3_O_4_/rGO does not exhibit such a uniform morphology distribution of Co_3_O_4_. In addition, we only found that a typical corrugated structure in Fig. 2D, suggesting there is no Co_3_O_4_ particles nucleate on N-rGO surface. The oxygen-containing functional groups of rGO were beneficial for the nucleation and anchoring of nanocrystals on the sheets to achieve covalent attachments, which help to shape the uniform formation of Co_3_O_4_^35^,^36^. In addition, these uniform structures of Co_3_O_4_ particles anchored into N-rGO can also be ascribed to NH_3_ together with oxygen-containing functional group coordinating with cobalt cations and thus reducing Co_3_O_4_ particles size and enhancing particles nucleation on N-rGO^37^. XRD was performed to investigate the phase structure of Co_3_O_4_/N-rGO. As shown in Fig. 3A, the diffraction peaks of the pristine Co_3_O_4_ was consistent with the standard Co_3_O_4_ (JCPDS card: 42-1467).

The major diffraction peaks of Co_3_O_4_/N-rGO were well in agreement with those of Co_3_O_4_ except for the broad (002) peak at approximately 25°, which can be ascribed to disordered stacked graphitic sheets^30^. This manifest that the original GO has been reduced to rGO during the hydrothermal process, again confirming we have successfully incorporated Co_3_O_4_ into rGO^38^. BET experiments was conducted to obtain specific surface area of as-prepared samples and the isotherms exhibit typical IV isotherms where the recorded BET surface area of Co_3_O_4_/N-rGO, N-rGO and Co_3_O_4_ are 103.9 m^2^/g, 139.7 m^2^/g, and 62.8 m^2^/g, respectively. These results indicate the change of N-rGO structure after doping with Co_3_O_4_.

Raman spectroscopy was carried out to extend the study for the carbon structures in Co_3_O_4_/N-rGO, N-rGO and Co_3_O_4_/GO hybrid which are shown in Fig. 3B, where the peaks of Raman spectrum of Co_3_O_4_ anchored on the N-rGO and Co_3_O_4_/GO hybrid at 193, 470 and 680 cm^−1^, can be attributed to the Eg, F2g and A1g modes of Co_3_O_4_^39^. It is noted that there are two remarkable peaks around 1339 and 1591 cm^−1^ refer to the D-band (arising from the edge or defect sites of carbon) and G band (representing the sp2 carbon) of the graphene domain, respectively^40^.

X-ray photoelectron spectroscopic (XPS) measurements were performed to determine the surface element constitution in Co_3_O_4_/N-rGO. The sharp peaks in Fig. 3C are corresponded to the characteristic peaks of C_1s_, O_1s_, N_1s_ and Co_2p_, indicating the existence of carbon, oxygen, nitrogen and cobalt elements in the prepared sample. The XPS spectrum for Co_2p_ shown in Fig. 3D reveals two major peaks with binding energies at 780.1 and 795 eV, corresponding to Co_2p3/2_ and Co_2p1/2_, respectively, with a spin energy separation of 15 eV, which is attributed to the Co^2+^ oxidation state, indicating that a portion of Co^3+^ is reduced to Co^2+^ with generating oxygen vacancies^17^. These results again confirmed that Co_3_O_4_ nanoparticles have been anchored on N-rGO, Co^2+^ and Co^3+^ in the crystal structure of Co_3_O_4_ are being considered to be playing a vital role in improving catalytic performance of oxygen reduction reaction and oxygen evolution reaction^37^. Furthermore, the main beautiful structure of Co_3_O_4_ is the peculiar cation distribution in the face centered cubic (FCC) crystal where the Co^2+^ ions reside on the 1/8^th^ of the tetrahedral A sites while the Co^3+^ ions occupy 1/2 of the octahedral B sites^11^, endow the system viable for electrocatalytic applications. The high-resolution N_1s_ XPS spectrum of Co_3_O_4_/N-rGO was used primarily to determine the bonding configurations of N atoms in the composite, as seen in Fig. 3E, The peak deconvolution suggests four components were centered at about 398, 400, 401, and 403 eV, corresponding to pyridinic N, pyrrolic N, quaternary N, and oxidized N, respectively. N atom have the lone electron pairs which can hybridize with sp^2^ carbon atoms to celebrate oxygen reduction reaction. The performance of ORR depends on the bonding configuration of N atoms in carbon materials. It has been reported that the onset potential of a nitrogen-doped catalyst has strong relation with pyridinic form nitrogen, but little effect by pyrrolic nitrogen and oxidized type nitrogen^41^.

### Electrocatalytic activity of Co_3_O_4_/N-rGO for H_2_O_2_ reduction

Though enzyme based electrochemical sensors have been widely developed to sensing H_2_O_2_ due to the advantages of high sensitivity and good selectivity, these sensors often suffer from unstable response due to the intrinsic nature of enzymes^42^. Therefore, it is necessary to develop a simple non-enzymatic strategy for sensing H_2_O_2_ with high sensitivity. To date, electrocatalysts for design H_2_O_2_ sensors with high sensitivity, good selectivity and easy regulation properties hold leading position among various sensors^12^. It has been proved that functional nano-structured transition-metal oxides exibit good electrocatalytic activity toward the H_2_O_2_ reduction, which provides valuable strategy for the nonenzymatic determination of H_2_O_2_^43^,^44^. Among various kinds of transition metal oxides, Co_3_O_4_ shows attracting electronic and electrocatalytic properties. Particularly, its normal spinel crystal structure is favorable for electron transportation between Co^2+^ and Co^3+^ ions and Co_3_O_4_ possess catalase-like activity, which is benefit to sensing H_2_O_2_. Therefore, Co_3_O_4_ have been extensively explored as the sensing materials for developing enzyme-free H_2_O_2_ sensors. However, Co_3_O_4_-based catalysts usually suffer from the poor electrical conductivity, low active site density and the dissolution or agglomeration during electrochemical processes^45^,^46^. On the other hand, graphene has the ability to promote electron transfer rates and graphene-based modified electrode had much better electrocatalysis toward H_2_O_2_^47^.

**Figure 4:**
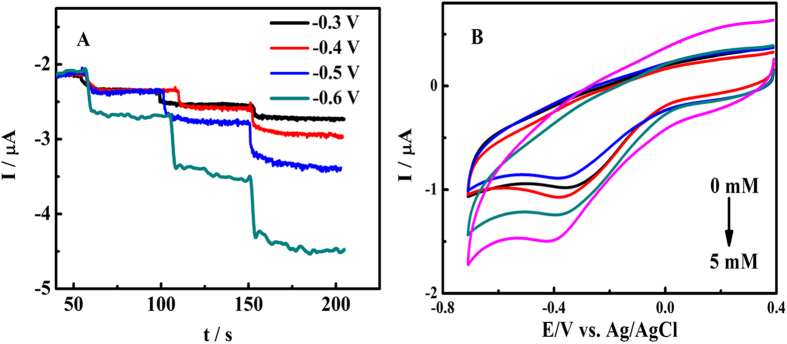
Cyclic voltammograms of (**A**) Co_3_O_4_/N-rGO, N-rGO and Co_3_O_4_/rGO modified GC electrodes in 0.1 M PB solution (pH 7.0) containing 5 mM H_2_O_2_ and (**B**) Co_3_O_4_/N-rGO modified GC electrode in 0.1 M PB solution (pH 7.0) containing different concentration of H_2_O_2_ (from the top: 0, 0.5, 1, 2 and 5 mM). Scan rate 50 mV s^−1^.

In our study, Co_3_O_4_ nanoparticles were incorporated into nitrogen doped graphene, leading to improved conductivity, enhanced catalytic activity and stability of the metal oxide nanocatalyst, and thus a better catalytic effect to H_2_O_2_ reduction due to the synergistic effect. To investigate the electrocatalytic characteristics to H_2_O_2_ reduction of Co_3_O_4_/N-rGO, voltammetric measurements were performed using the Co_3_O_4_/N-rGO, Co_3_O_4_/rGO and N-rGO modified GC electrodes in the presence of 5 mM H_2_O_2_ at a scan rate of 0.05 V s^−1^. Figure 4A exhibit that a distinct catalytic current peak at −0.40 V could be ascribed for Co_3_O_4_/N-rGO modified GC electrode. Figure 4B reveals the CVs of Co_3_O_4_/N-rGO modified GC in the presence of different concentration of H_2_O_2_ in 0.1 M PB solution (pH7.0) at the scan rate of 50 mV s^−1^. It demonstrates that the reduction current gradually increases with the increase of H_2_O_2_ concentration (from the top: 0.5 1, 2 and 5 mM), which manifest the Co_3_O_4_/N-rGO material have improved electrocatalytic activity to H_2_O_2_ and pave a route for quantitive analysis.

**Figure 5:**
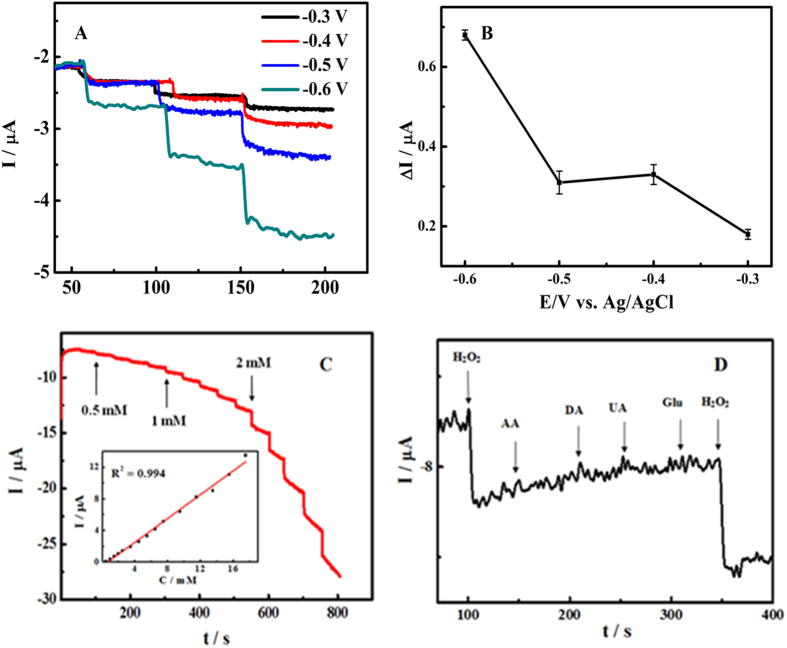
The effect of applied potential (**A**,**B**) to the amperometric response of sequential addition of 2 mM H_2_O_2_ on the Co_3_O_4_/N-rGO modified GC electrode. Amperometric response of Co_3_O_4_/N-rGO to successive addition of H_2_O_2_. The inset is the plot of H_2_O_2_ peak current versus H_2_O_2_ concentration (**C**). Amperometric response of the Co_3_O_4_/N-rGO exposed to H_2_O_2_, AA, DA, UA and glucose. Applied potential: −0.6 V and supporting electrolyte: 0.1 M PB solution (pH 7.0) (**D**).

It is a significant way for amperometric technique to test the sensing property of Co_3_O_4_/N-rGO modified GC electrode. We studied the effect of the applied potential in order to improve the Co_3_O_4_/N-rGO modified GC electrode performance towards non-enzymatic H_2_O_2_ sensing. We investigated applied potential on the amperometric response on the Co_3_O_4_/N-rGO modified GC electrode towards sequential addition of 0.5 mM H_2_O_2_ by varying the potential between−0.6 V and −0.3 V. As shown in Fig. 5A and B, as amperometric response of the Co_3_O_4_/N-rGO modified GC electrode has the optimal sensitivity, the applied potential at −0.6 V was selected. Figure 5A shows a typical current-time plot of the Co_3_O_4_/N-rGO modified GC electrode on successive addition of H_2_O_2_ at an applied potential of −0.6 V. Catalytic currents showed linear response to H_2_O_2_ from 0.5 mM to 17.5 mM (R^2^ = 0.994) with a detection limit (S/N = 3) to be 0.1 mM, which are comparable to or even better than those of the other metal-free or enzyme based H_2_O_2_ biosensors^9^,^32^,^48^. To investigate the selectivity for H_2_O_2_ sensing, the amperometric responses of ascorbic acid (AA), dopamine (DA), uric acid (UA) and glucose (Glu) are investigated on the Co_3_O_4_/N-rGO modified GC electrode. As shown in Fig. 5D, when the Co_3_O_4_/N-rGO-modified GC electrode was polarized at −0.6 V, the addition of 0.2 mM AA, 0.02 mM DA, 0.2 mM UA and 5 mM Glu did not produce an observable current response while the addition of H_2_O_2_ induced obvious reduction currents reponse, indicating that the measurements of H_2_O_2_ are essentially interference-free from other relevant electroactive species. Therefore, the as-prepared the Co_3_O_4_/N-rGO modified GC electrode is a good candidate for the fabrication of stable and specific amperometric sensor for the nonenzymatic detection of H_2_O_2_. The excellent performance of H_2_O_2_ sensor can be ascribed to the well distributed and high loading amount of Co_3_O_4_ nanoparticles.

### The performance of oxygen reduction reaction

**Figure 6:**
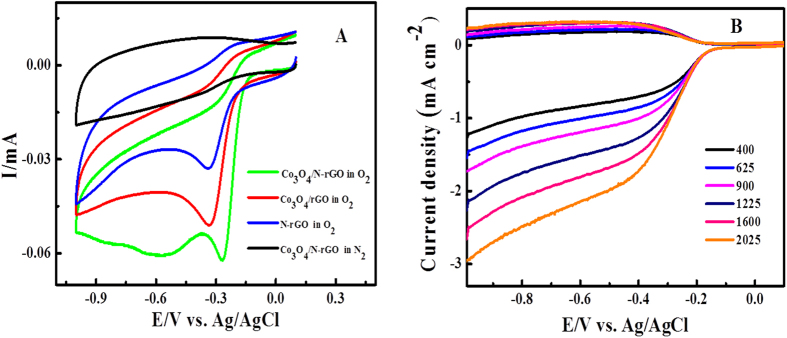
(**A**) Cyclic voltammograms of Co_3_O_4_/N-rGO, Co_3_O_4_/rGO, N-rGO modified GC electrodes in an O_2_-saturated and in N_2_-saturated 0.1 M KOH at a scan rate of 10 mV s^−1^. (**B**) LSVs of Co_3_O_4/_N-rGO on RRDE in 0.1 M KOH with various rotation rates at a scan rate of 5 mV s^−1^.

To evaluate the ORR catalytic activity of Co_3_O_4_/N-rGO, N-rGO and Co_3_O_4_/rGO, CV measurements were performed in both O_2_ and N_2_-saturated 0.1 M KOH solution. As shown in Fig. 6A, CV of N-rGO and Co_3_O_4_/rGO in the O_2_-saturated electrolyte shows a reduction peak at −0.34 V and −0.33 V respectively, suggesting their electrochemical catalytic activity for ORR. As for Co_3_O_4_/N-rGO composite modified electrode, a reduction peak at ca. −0.26 V is observed, which is more positive than those of N-rGO and Co_3_O_4_/rGO while it also has a highest current density, suggesting a great improvement of catalytic activity, which is better than tri-functional carbon materials in previous study^32^. Previous have reported that the electrocatalytic activity of Co_3_O_4_ was mainly affected by structure^46^. Co_3_O_4_ particles have a spinel structure and the direct Co-Co interactions across shared octahedral edges of its spinel framework can enhance the electronic conductivity which is beneficial to the ORR catalytic activity. On the other hand, the N-graphene also exhibited a much better electrocatalytic activity, long-term operation stability for oxygen reduction reaction^22^. Therefore, such an excellent electrocatalytic activity of the Co_3_O_4_/N-rGO toward ORR can be ascribed to the synergetic chemical coupling effects of Co_3_O_4_ and N-graphene^18^,^49^,^50^.

To investigate the oxygen reduction mechanism of Co_3_O_4_/N-rGO modified GC electrodes, the ORR was studied by RRDE technique via measurement of the yield of the generated intermediate H_2_O_2_. The RRDE technique was applied to quantitatively determine the n value toward ORR and the H_2_O_2_ generation rate by setting the potential of the ring electrode at 0.4 V.

The electron transfer number n of ORR and HO_2_^−^ intermediate production percentage (HO_2_^−^ %) were determined as


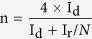



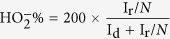


where I_d_ is the disk current, I_r_ is the ring current, and N is the current collection efficiency of the Pt ring, which was determined to be 0.4^21^,^46^. From the Fig. 6B, it was calculated H_2_O_2_% value for the Co_3_O_4_/N-rGO during ORR process is about 63.5–32.2% at potentials ranging from −0.3 to −0.8 V. The calculated n value for the Co_3_O_4_/N-rGO is about 2.9 to 3.4 from −0.3 to −0.8 V. These results reveal that the electrocatalytic process of Co_3_O_4_/N-rGO is an improved four-electron pathway and a two-electron transfer pathway occurred simultaneously for ORR.

**Figure 7:**
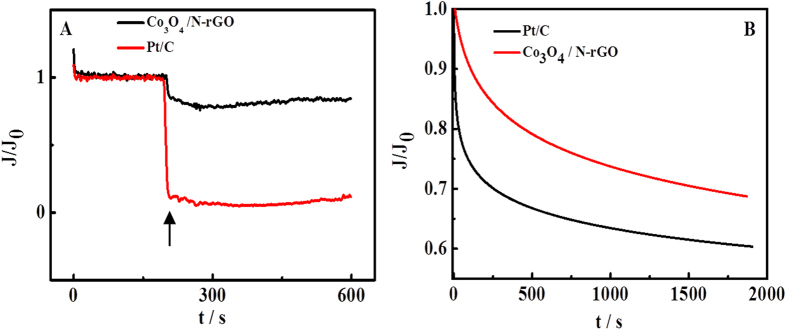
(**A**) Chronoamperometric responses of Co_3_O_4_/N-rGO and Pt/C (20%) at −0.3 V in the O_2_-saturated 0.1 M KOH. The arrow indicates the addition of 3.0 M methanol into the O_2_-saturated electrochemical cell. (**B**) Chronoamperometric responses obtained at the Pt/C (20%) and Co_3_O_4_/N-rGO at −0.3 V in O_2_-saturated 0.1 M KOH.

Methanol poisoning and stability are key issues challenging the cathode materials in current fuel cell techniques^51^. The effect of methanol poisoning and stability on the Co_3_O_4_/N-rGO was investigated in Fig. 7A and B by current-time (i-t) chronoamperometry. As shown in Fig. 7A, when methanol was injected, a significant decrease (90.7%) in current was observed for the Pt/C electrode, whereas only a slight decrease (13.3%) was observed for the Co_3_O_4_/N-rGO, suggesting poor tolerance of Pt/C to methanol compared with the Co_3_O_4_/N-rGO material. Figure 7 Bshows that the amperometric response of ORR on the Co_3_O_4_/N-rGO which exhibits a very slow attenuation of relative current, after 2000 s i.e. a current loss of approximately 31.37%. In contrast, the Pt/C reveals degraded stability with a current loss (39.58%) after 2000 s, indicating the Co_3_O_4_/N-rGO has a better stability than Pt/C.

### The catalytic property of oxygen evolution reaction

**Figure 8:**
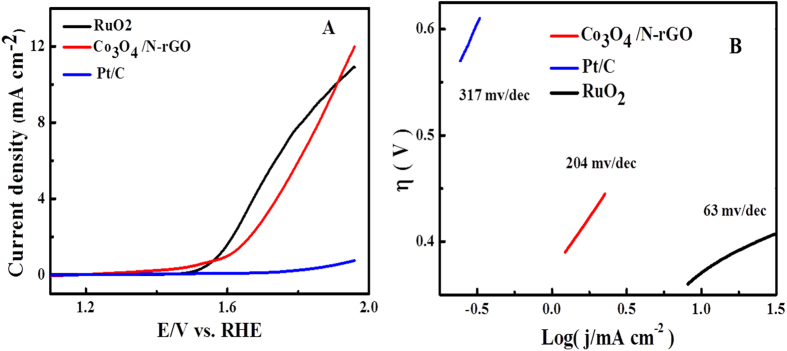
(**A**) The OER polarization curves of Co_3_O_4_/N-rGO catalyst and commercial Pt/C (20%) at a sweep rate of 5 mV s^−1^ using RDE with a rotation speed of 1600 rpm and (**B**) corresponding Tafel plots.

Previous studies have reported Co_3_O_4_ particles deposited on stable supporting and conducting substrates can be used as effective electrode materials for both the oxygen reduction (ORR) and evolution (OER) reactions via decreasing overpotential in fuel cells and water electrolyzers^17^,^52^. The good catalytic performance of OER can be ascribe to the small crystalline size and the mixed valences Co^2+^ and Co^3+^ of Co_3_O_4_ as well with conductive support substrates^17^. Sun and his groups synthesize Co_3_O_4_ nanorod–multiwalled carbon nanotube hybrid with a onset potential of about 0.47 V vs. Ag/AgCl and Tafel slope of 65 mV/dec^37^. In our work, a rotational disk electrode (RDE) tests were also carried out in alkaline solution to further evaluate the OER catalytic activity of the Co_3_O_4_/N-rGO. Figure 8A showed the typical linear sweep voltammograms using the RDE at an electrode rotating speed of 1600 rpm and a potential scanning rate of 5 mV s^−1^. From the OER region, the Co_3_O_4_/N-rGO afforded a sharp onset potential at 1.54 V, which is worse than that of RuO_2_ at 1.49 V and better than Pt/C. The OER over potential at current density of 10 mA cm^−2^ is close to that of RuO_2_, indicating the Co_3_O_4_/N-rGO has a good OER property. The Tafel slope is usually used to study the catalytic mechanism of electrocatalysis for OER. In Fig. 8B, The Tafel slope comparison showed that Co_3_O_4_/N-rGO has Tafel slope of 204 mV/dec which is much smaller than those for Pt/C (308 mV/dec) and is bigger than RuO_2_ (63 mV/dec), suggesting Co_3_O_4_/N-rGO has an improved performance of OER.

## Conclusions

In summary, this study describes a facile and effective route to synthesize hybrid material consisting of Co_3_O_4_ nanoparticles anchored on nitrogen-doped reduced graphene oxide (Co_3_O_4_/N-rGO) as a high-performance tri-functional catalyst for ORR, OER and H_2_O_2_ sensing. Owing to the synergetic chemical coupling effects between Co_3_O_4_ and graphene, the Co_3_O_4_/N-rGO exhibited excellent electrocatalytic activity with a direct reduction to H_2_O_2_ at −0.6 V and sensing ability towards H_2_O_2_. Although Co_3_O_4_/rGO or N-rGO alone has little catalytic activity, the Co_3_O_4_/N-rGO exhibits high ORR activity with ORR peak potential to be −0.26 V (vs. Ag/AgCl) and the number of electron transfer number is 3.4, excellent tolerance to methanol crossover and exceptionally good stability to Pt/C (20%) in alkaline solutions. Catalytic studies of Co_3_O_4_/N-rGO for OER display a better onset potential, overpotential under the current density of 10 mA cm^−2^ and a smaller Tafel slope with Pt/C (20%). Due to the ease of synthesis and electrode fabrication, the method developed by this study could be used for large-scale synthesis of non-precious metal-based trifunctional metal catalyst for hydrogen peroxide reduction, ORR and OER.

## Experimental

### Chemicals and materials

Nafion perfuorinated resin solution (5 wt% in a mixture of lower aliphatic alcohols and water) and commercial platinum/carbon (Pt/C) 20 wt% (Pt loading: 20 wt%, Pt on carbon black) were obtained from Sigma-Aldrich. All other chemicals (analytical grade) were purchased from Beijing Chemical Reagent Company (Beijing, China) and used without further purification. Ultra-pure water was obtained with a Milli-Q plus water purification system (Milli-pore Co. Ltd., USA).

### Materials characterization

Scanning electron microscopy (SEM) images were obtained on a Hitachi S-2600N scanning electron microscope. Elemental analysis data were obtained through Flash EA 1112. The X-ray photoelectronspectra (XPS) spectra were obtained using a VG Micro-tech ESCA 2000 using a monochromic 15 Al X-ray source. For rotating ring-disk electrode (RRDE) measurements, a bipotentiostat (CHI 832, Shanghai Chenhua Instrument Co. Ltd.) and a rotating ring-disk electrode with a rotating GC disk electrode and a platinum ring electrode (ALS RRDE-2) were used. The collection efficiency of the ring-disk electrode was evaluated with the Fe(CN)_6_^3−^/^4−^ redox couple and was calculated to be 0.4. Electrochemical measurements were performed with a computer-controlled Electrochemical analyzer (CHI600E, Chenhua, China) in a two-compartment electrochemical cell with as-prepared material modified on a glassy carbon electrode (3 mm in diameter) as working electrode, a platinum wire as counter electrode, and a Ag/AgCl (3 M KCl) electrode as reference electrode. All electrochemical experiments were performed at room temperature.

### Preparation of Co_3_O_4_/N-rGO, N-rGO and Co_3_O_4_/rGO materials

16.5 mg oxidized graphene oxide (GO) were redispersed in 50 mL anhydrous ethanol to form GO anhydrous ethanol suspension with concentration to be 0.33 mg/mL. The first step to prepare Co_3_O_4_/N-rGO was performed by adding 3.6 ml of 0.2 M Co(Ac)_2_ aqueous solution to 72 ml of GO anhydrous ethanol suspension, followed by the addition of 1.8 ml of NH_4_OH (30% solution) and 2.1  ml of water, consequently. The reaction was kept at 80 °C with stirring for 10 h. After that, the reaction mixture from the first step was transferred to a 100 mL autoclave for hydrothermal reaction at 180 °C for 12 h. Co_3_O_4_/GO hybrid was made by the same steps without adding NH_4_OH (30% solution) in the first step^26^. N-rGO hybrid was also made by the same steps just as making Co_3_O_4_/N-rGO preparation without adding Co(Ac)_2_ aqueous solution.

### The fabrication of as-prepared materials modified electrodes

A rotating ring-disk electrode (RRDE) with a rotating glassy carbon (GC) disk electrode (4 mm diameter) and a platinum ring electrode (ALS RRDE-2), and a GC electrode with a diameter of 3 mm working electrode were used as working electrode in this study. Prior to the surface modification, the delectrode were polished with 1.0, 0.3, and 0.05  μm alumina slurries, and finally rinsed with Milli-Q water under an ultrasonic bath for 1 min. A Co_3_O_4_/N-rGO modified GC electrode was prepared by casting the 4 μL of 2 mg/mL Co_3_O_4_/N-rGO suspension on the disk electrode surface and drying in air to evaporate the solvent. Similarly, 4 μLof 2 mg/mL N-rGO solution and 4 μL of 2 mg/mL Co_3_O_4_/GO suspension were dropped on GC electrodes, respectively and dried in air to evaporate the solvent for control experiment. Finally, 5 μL nafion (0.5%) solution (diluted 10 times with deionized water) was covered onto electrode surface and dried to form modified working electrode.

All of the electrochemistry experiments were performed at room temperature. The Co_3_O_4_/N-rGO modified GC electrode was pretreated by electrochemical oxidation in a phosphate buffered solution (pH =6.8) at a potential of 1.7 V (vs. Ag/AgCl) for 300 s at room temperature, followed by potential sweeping from 0.0 V to 1.4 V in 0.5 M H_2_SO_4_ until a stable voltammogram was achieved, the purpose of electrochemical oxidation in phosphate buffered solution and H_2_SO_4_ is increased more oxygen containing functional group in carbon materials to increase the active site in oxygen reduction reaction. some Co_3_O_4_ nanoparticles may dissolv in H_2_SO_4_ thus leaves more active sites on grapheme. For linear sweep voltammetry (LSV) from 0.2 to −1.0 V, The Co_3_O_4_/N-rGO modified GC was scanned at a scan rate of 10 mV·s^−1^ to measure the surface behavior of the ORR activity of the catalyst in O_2_-saturated 0.1 M KOH. For more quantitative measurements of the ORR activity, LSV was conducted on the catalyst-coated RRDE at a scan rate of 5 mV·s^−1^ in O_2_-saturated KOH solution at various rotation rates from 400 to 2025 r·min^−1^.

## Additional Information

**How to cite this article:** Zhang, T. *et al*. Co_3_O_4_ nanoparticles anchored on nitrogen-doped reduced graphene oxide as a multifunctional catalyst for H_2_O_2_ reduction, oxygen reduction and evolution reaction. *Sci. Rep.* **7**, 43638; doi: 10.1038/srep43638 (2017).

**Publisher's note:** Springer Nature remains neutral with regard to jurisdictional claims in published maps and institutional affiliations.

## Figures and Tables

**Figure 1:**
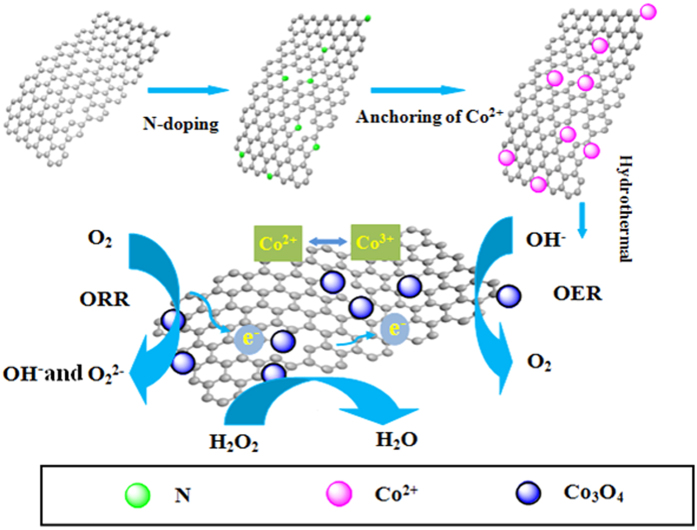
Schematic illustration of the synthesis of Co_3_O_4_/N-rGO.
